# Evaluation of Drug–Polymer and Drug–Drug Interaction in Cellulosic Multi-Drug Delivery Matrices

**DOI:** 10.3390/mps8010004

**Published:** 2025-01-06

**Authors:** Abdullah Isreb, Mohamed A. Alhnan, Abdulrahman Mkia, Khaled Al-Jammal, Abdallah M. Yaghi, Enoche Florence Oga, Peter Timmins, Michael Bonner, Robert T. Forbes

**Affiliations:** 1 Department of Clinical Sciences, Liverpool John Moores University, Liverpool L3 3AF, UK; 2 Department of Life Science and Medicine, Kings College University, London SE1 9NH, UK; alhnan@kcl.ac.uk; 3 Department of Pharmacy, Al-Ahliyya Amman University, Amman 19111, Jordan; a.mkia@ammanu.edu.jo; 4 Quay Pharma Ltd., Deeside Industrial Park, Quay House, 28 Parkway, Deeside CH5 2NS, UK; khaled.aljammal@sgs.com; 5 Department of Information, University of Sheffield, Sheffield S10 2AH, UK; a.m.yaghi@sheffield.ac.uk; 6 Department of Pharmacy and Biomedical Sciences, University of Central Lancashire, Preston PR1 2HE, UK; eoga@uclan.ac.uk (E.F.O.); rtforbes@uclan.ac.uk (R.T.F.); 7 Department of Pharmacy, University of Huddersfield, Huddersfield HD1 3DH, UK; p.timmins@hud.ac.uk; 8 Department of Life Sciences, University of Bradford, Bradford BD7 1DP, UK; superbarriercream@hotmail.co.uk

**Keywords:** Hansen solubility parameters, solid dispersion, multi-drug delivery system, drug interaction, substitution in solid matrix, drug–polymer interaction, HPMCAS, carboxylic acid, pharmaceutics

## Abstract

Multi-drug delivery systems have gained increasing interest from the pharmaceutical industry. Alongside this is the interest in amorphous solid dispersions as an approach to achieve effective oral delivery of compounds with solubility-limited bioavailability. Despite this, there is limited information regarding predicting the behavior of two or more drugs (in amorphous forms) in a polymeric carrier and whether molecular interactions between the compounds, between each compound, and if the polymer have any effect on the physical properties of the system. This work studies the interaction between model drug combinations (two of ibuprofen, malonic acid, flurbiprofen, or naproxen) dispersed in a polymeric matrix of hypromellose acetate succinate (HPMCAS) using a solvent evaporation technique. Hildebrand and Hansen calculations were used to predict the miscibility of compounds as long as the difference in their solubility parameter values was not greater than 7 MPa^1/2^. It was observed that the selected APIs (malonic acid, ibuprofen, naproxen, and flurbiprofen) were miscible within the formed polymeric matrix. Adding the API caused depression in the Tg of the polymer to certain concentrations (17%, 23%, 13%) for polymeric matrices loaded with malonic acid, ibuprofen, and naproxen, respectively. Above this, large crystals started to form, and phase separation was seen. Adding two APIs to the same matrix resulted in reducing the saturation concentration of one of the APIs. A trend was observed and linked to Hildebrand and Hansen solubility parameters (HSP).

## 1. Introduction

Polymers are an integral part of many drug delivery systems. They can be used in tablet coating [1], as binders in tablet formulations [2], bulking agents [3], among others. Moreover, polymers have been used in the manufacture of nanomaterials, whether polymeric nanoparticles [4,5,6], dendrimers [7], or other forms of delivery [8]. Polymeric materials such as cellulose ethers and polyvinyl alcohol are typically used as the basis of amorphous solid dispersions. When dispersing a drug into a polymeric matrix, it is either miscible or not. Moreover, it was noted that even for drugs that were dispersed in polymeric matrices, crystal growth was inhibited to various degrees using different polymers [9,10]. However, to the best of the authors’ knowledge, there is no research that has studied the effect of multiple components on the behavior and interaction within the formed matrices. Most related research focuses on the interaction of single components with a single polymer in the formed matrix. Whilst it is not unusual to have multiple drugs included together in an oral solid dosage form, it seems to be uncommon to find more than one drug, or a drug and an additional non-polymeric agent combined and dispersed in a single polymeric matrix to form a combination amorphous solid dispersion. There are fixed-dose combinations based on amorphous solid dispersions, but these contain individual co-formulated drug dispersions. Hence, understanding the effect of adding multiple components on the behavior and interaction between a polymer and multi-components is deemed important.

Solubility and miscibility of materials are important not only to pharmaceutical formulations but also to other industries, with the miscibility of compounds believed to be linked to their chemical properties. Hildebrand and Scott developed a theory in 1949 to relate chemical properties of compounds to their behavior upon mixing, referred to as solubility parameters or total cohesion parameters. These parameters were investigated and updated to meet the demand of developing and improving paints as well as to determine the best solvent system for various compounds [11]. Observations from those studies revealed differences in drying behavior between paints made with various solvents. The solubility parameter addresses these issues and links the physicochemical properties of compounds with their behavior and affinities. Solubility parameters can be defined as the square root of cohesive energy density (cohesive energy per unit volume) of the compound [12], and Hildebrand related the energy of mixing to the energy of vaporization of the raw materials [3]. The Hildebrand solubility parameter, also known as the total cohesion parameter, can be calculated using the following equation (Equation (1) [12] Hildebrand and Scott solubility parameter equation):

(1) $$\delta ={\left(C.E.D\right)}^{0.5}={\left(\frac{-U}{V}\right)}^{0.5}$$

where *C.E.D* is the cohesive energy density, *U* is the molar internal energy, and *V* is the molar volume.

The theory of Hildebrand was developed for nonpolar and non-associating systems [12]. However, since the effect of hydrogen bonding on the interaction and the general behavior of polar compounds is important and cannot be ignored, the theory was later modified by other scientists such as Prausnitz [13,14] to include other factors to make the theory applicable to polar compounds as well. Nevertheless, for certain compounds, the theoretical values of solubility parameters calculated by that equation did not match the practical values obtained using inverse gas chromatography. Therefore, Hansen expanded the theory of Hildebrand and added what is known as the three-dimensional solubility parameters, which relate to the effect of hydrogen bonding, polar forces, and dispersion forces [11]. The theory of solubility parameter was extrapolated later to include the miscibility of liquid as well as solid materials [15,16].

Additional factors were considered later and added to the equation, such as the group contribution value of each group in the molecule. These allowed more precise calculation of the solubility parameter values and less variation between the calculated and the measured values (using inverse gas chromatography). Therefore, another equation was developed to calculate the solubility parameter values based on their group molar cohesive energy and molar vaporization energy (Equation (2) [12] Solubility parameter equation using group contribution method).

(2) $$\delta ={\left[\frac{\sum z\;\left(Z_{u}\right)}{\sum z\;\left(Z_{v}\right)}\right]}^{0.5}$$

where *z* represents the contributing groups.

Hildebrand and Hansen solubility parameters were utilized to predict the miscibility of many solid dispersion systems composed of a polymer and a single compound (binary system) [15,16,17,18,19,20]. However, to the best of the authors’ knowledge, there are no available data in the literature about the miscibility/interactions between two or more compounds in a polymeric matrix (with the polymer or with each other). Multiple drug delivery systems are becoming more and more popular, especially in formulations used in therapy for cardiovascular, metabolic disease, anti-cancer, and anti-infection/inflammation [19,20,21,22,23,24,25], hence, the growing need to study the stability of such combinations. Such drugs, formulated as multiple drug medications, can be added in multi-layer tablets [25] or can be added together [26]. Hansen/Hildebrand solubility parameters (HSP) predict the total miscibility of these systems (drugs and polymers), hence the amorphous nature of both drugs in the polymeric matrix. This study aimed to investigate the use of the solubility parameter to predict the solubility/miscibility of mixtures of drugs in a polymeric matrix.

The aim of this study is to assess combinations of drugs in a polymeric matrix and identify trends to enable the prediction of which one of the two drugs will be miscible in the polymeric matrix and which one will separate (phase out) and form a crystalline phase.

## 2. Experimental Design

### Materials

1. Malonic acid (Sigma-Aldrich, Gillingham, UK, Cat. no.: 792535), Reagent Plus 99%
2. Ibuprofen (Sigma-Aldrich, Gillingham, UK, Cat. no.: I4883), >98% GC grade
3. Naproxen (Sigma-Aldrich, Gillingham, UK, Cat. no.: N8280), USP testing specification
4. Flurbiprofen Sigma-Aldrich, Gillingham, UK, Cat. no.: F8514), ≥98.5%
5. HPMCAS (Shin-Etsu, Japan, MG grade, Cat. no.: AQOAT AS-MG)

Solvents used:

6. Acetone (Fisher Scientific, Loughborough, UK, Cat. no.: 13277983)
7. chloroform (Fisher Scientific, Loughborough, UK, Cat. no.: 11398187)

All solvents used were of HPLC grade.

## 3. Procedure

Film casting by the solvent evaporation method [18] was adapted to prepare films containing single components and dual components as follows:

Malonic acid, ibuprofen, flurbiprofen, or naproxen were dissolved in acetone/chloroform 3:2 *v*/*v*. HPMCAS was added gradually while stirring until fully dissolved. The solution was cast in a plate and dried in a fume hood at room temperature. Films were dried for at least one week in order to ensure the complete removal of unbound moisture. Prepared concentrations are summarized in Table 1.

For polymeric matrices with a binary system (two drugs), various concentrations were dissolved in the solvent mixture (acetone/chloroform). Once both drugs were fully dissolved, the polymer was added in a similar pattern to the previous step, the solution was cast onto plates, and then left to dry under room temperature and pressure.

Solubility parameter values for each of the drugs and the polymer were calculated using Equation (2), and the values are presented in Table 2.

### 3.1. Characterization of the Drug–Polymer Mixtures

#### 3.1.1. Thermogravimetric Analysis (TGA)

A TA Instruments (Elstree, UK) Q5000 thermogravimetric analysis (TGA) was used to measure the thermal degradation profile for drugs, polymers, and cast polymeric matrices. Samples of 10 mg were loaded into an aluminum pan, which was then loaded into platinum pans. The samples were heated from room temperature to 400 °C at a rate of 10 °C/min. Heating was performed under a nitrogen purge of 40 mL/min, and data were analyzed using TA Universal analysis software v4.5a.

#### 3.1.2. Differential Scanning Calorimetry (DSC)

A TA Instruments (Elstree, UK) Q2000 differential scanning calorimetry (DSC) was used to measure the glass transition temperature and the melting endotherms of polymers and drugs. Sample of drugs, polymer, and stamps of dried films (using cork porer) of about 5 mg were placed in Tzero pans with pin-holed lids. Samples were subjected to a heat/cool/heat run at 10 °C/min. Samples were heated to 100 °C, held isothermal for 5 min, cooled to 0 °C, held isothermal for 2 min, and finally heated to 120 °C. All samples were measured in triplicate. Samples were measured under nitrogen gas purge of 50 mL/min.

#### 3.1.3. Powder X-Ray Diffraction (PXRD)

X-ray diffraction patterns were obtained on a Bruker D8 X-ray diffraction system (Bruker Corporation, Bruker AXS, Cambridge, UK). Samples were scanned in continuous mode from 3° to 50° (2θ) using a 0.01° step width and a 1 s time count. The receiving slit was 1° and the scatter slit 0.2°. The wavelength of the X-ray was 0.154 nm using a Cu source. The voltage used was 40 kV, and filament emission was 30 mA.

#### 3.1.4. Hot Stage Microscope (HSM)

Films were scanned using a bright field Zeiss hot stage microscope (Thornwood, NY, USA) equipped with an Axiocam MRC 5 Zeiss, Tv2/3”c, 0.63x, 1069-414 camera. The stage was connected to a heating unit (Linkam, Guildford, Surrey, UK). Cross-polarized light was used to identify the crystals inside the films. Samples were heated at a heating rate of 5 °C/min. Images were obtained when any change was observed.

## 4. Results and Discussions

**Figure 1:**
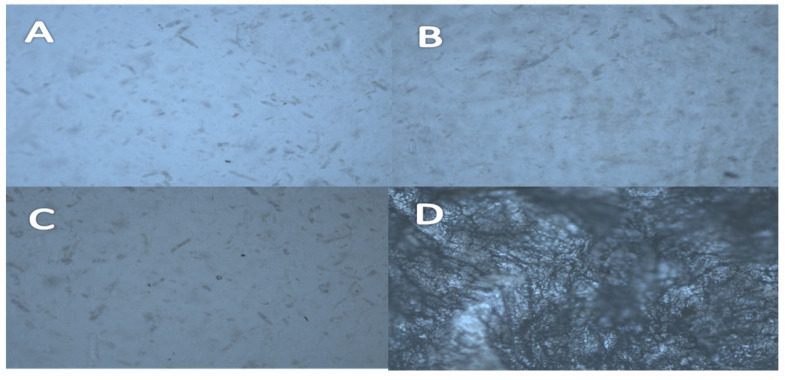
HSM analysis of films containing (**A**) 9%, (**B**) 13%, (**C**) 17%, and (**D**) 33% malonic acid in HPMCAS matrices. A magnification power of 100× was used.

**Figure 2:**
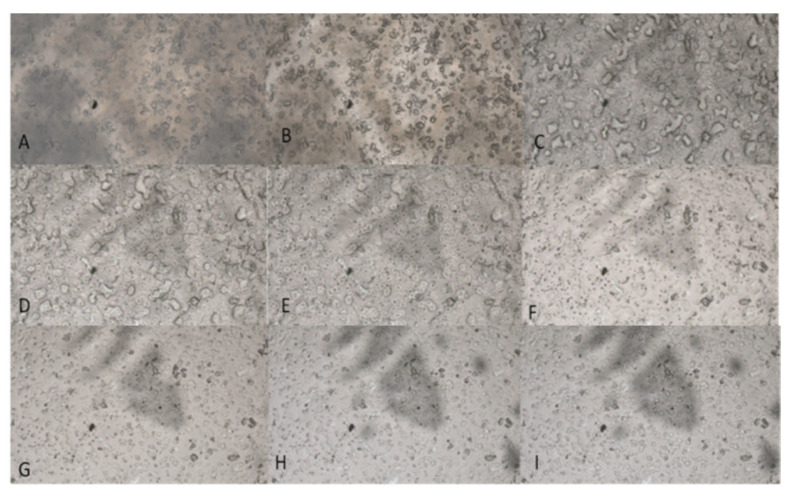
HSM analysis of films containing 19% malonic acid film at (**A**) 62 °C, (**B**) 65 °C, (**C**) 75 °C, (**D**) 76 °C, (**E**) 79 °C, (**F**) 83 °C, (**G**) 100 °C, cooling (**H**) 50 °C, reheating (**I**) 80 °C. Magnification of 100× was used.

Visual inspection of the polymeric matrices with a single drug revealed a haziness in the matrices that increases with increasing concentration of drugs. Polymers with low drug loading showed clear matrices. Hot stage microscope images revealed cylinder/spike-like shapes for malonic acid crystals (Figure 1). They appeared to melt at 134 °C when heated on the hot stage. It was observed that the molten crystal diffused through the polymeric matrix and did not recrystallize once the temperature was lowered again (Figure 2).

**Figure 3:**
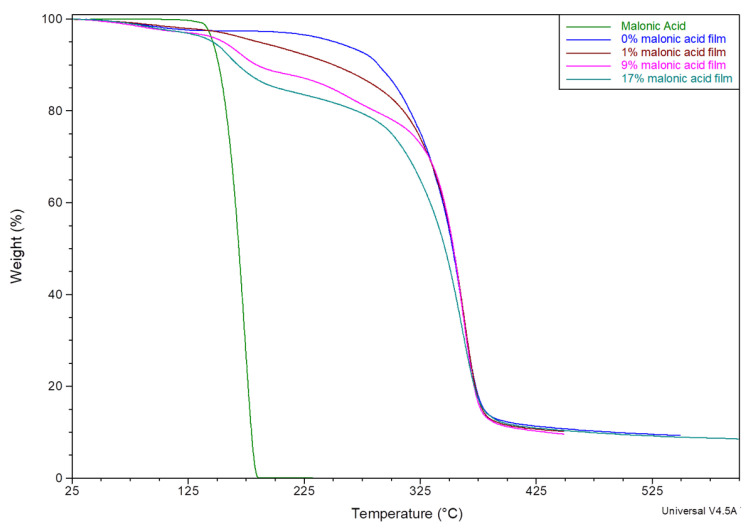
TGA thermal decomposition profiles for malonic acid-loaded polymeric matrices with concentrations of 1% (green), 9% (blue), and maroon (17%).

**Figure 4:**
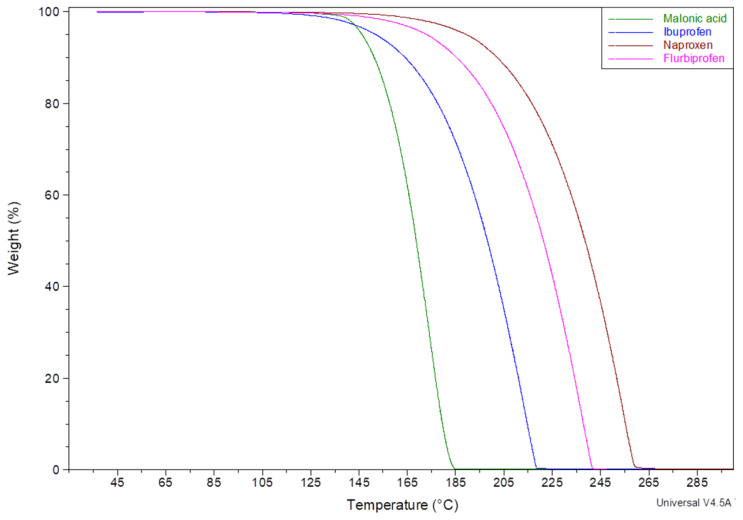
TGA Thermal decomposition profile of pure malonic acid, ibuprofen, naproxen, and flurbiprofen.

**Figure 5:**
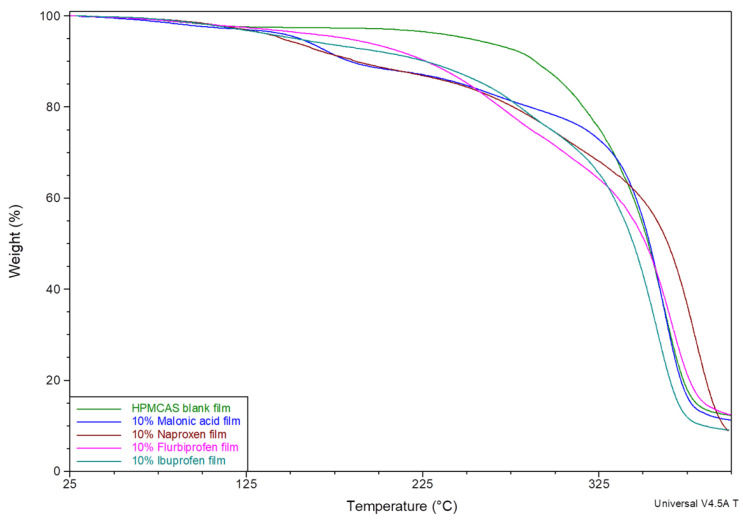
TGA thermal decomposition profiles for polymeric matrices loaded with 10% naproxen, flurbiprofen, and ibuprofen.

Thermal decomposition of these matrices did not show a marked moisture loss. Thermal profiles revealed about 3% weight loss till around 120 °C, which can be attributed to moisture. All matrices showed similar moisture content, which is equivalent to that of a pure HPMCAS cast matrix (Figure 3). The second stage of degradation started at about 150 °C and is believed to be the degradation of malonic acid, as it matches the thermal degradation profile of pure malonic acid (Figure 4). The increase in weight loss matches the concentration of malonic acid added to each matrix. The same trend was noticed with polymeric matrices loaded with ibuprofen, naproxen, or flurbiprofen (Figure 5).

Thermal degradation of malonic acid showed no moisture loss at about 120 °C. However, the moisture content of polymer without malonic acid still revealed a moisture content of about 3%, similar to all other polymeric matrices loaded with various concentrations of malonic acid.

Additionally, it was noticed for films containing 10% ibuprofen, naproxen, or flurbiprofen that they all also present moisture content of about 3–4% when tested using TGA (Figure 5). Hence, films were confirmed to be properly dried before tested further to guarantee the accuracy of the data and eliminate the solvent effect as another variable in the comparison. Data are provided within the Supplementary Data.

Additionally, the TGA thermographs of polymeric matrices containing various concentrations of naproxen and flurbiprofen were measured and revealed a similar pattern of moisture loss at 120 °C (about 3–5% for naproxen and 2–4% for flurbiprofen) (Figures S1 and S2). Moreover, polymeric matrices containing a combination of flurbiprofen and malonic acid showed a similar moisture loss (Figure S3), which eliminates the likelihood of moisture impact on the interaction between the drug and the polymeric matrix as it was described by Stefanie et al. [27]. 

**Figure 6:**
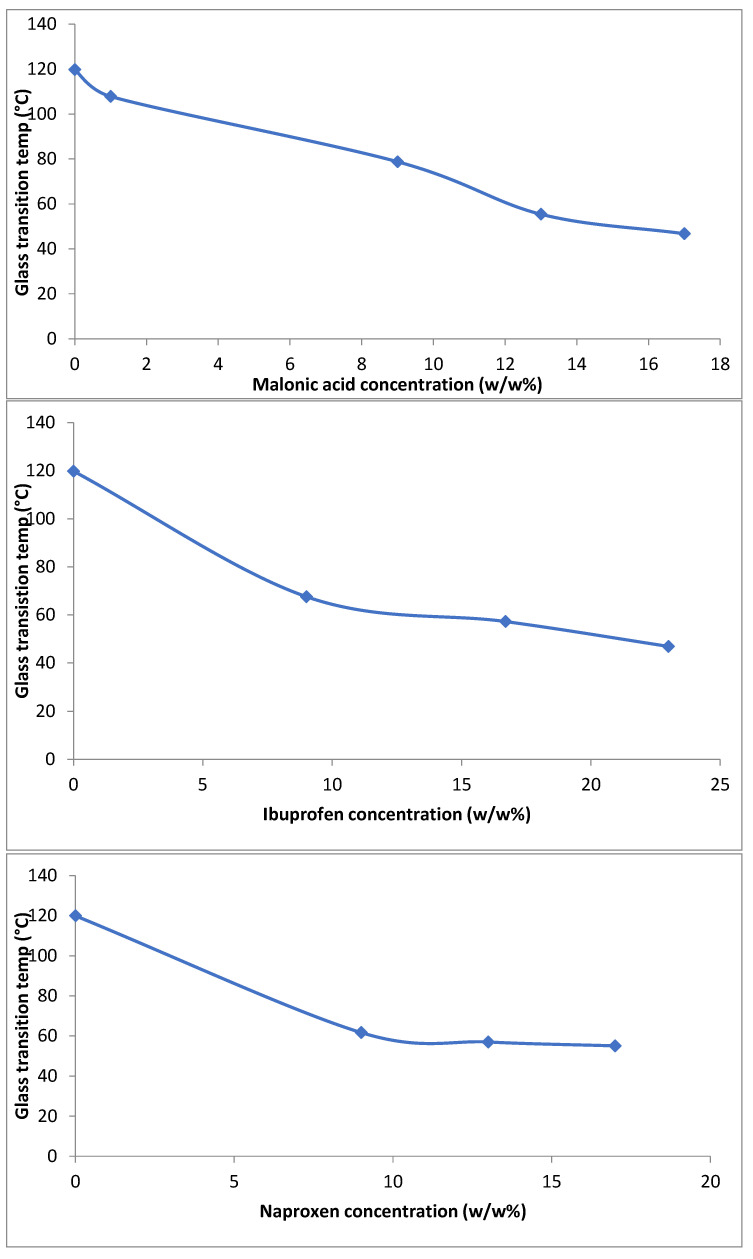
Representation of Tg vs. concentration of malonic acid (**top**), ibuprofen (**middle**), and naproxen (**bottom**) in HPMCAS matrices.

It was observed that the plasticity of the polymeric matrices increased with increasing the drug concentration in the matrix, confirmed through the glass transition temperature (Tg) of the polymeric matrices. It was shown (Figure 6) that drugs were depressing the measured Tg of the raw HPMCAS (from 119.97 °C) to much lower temperatures, which appeared to increase the plasticity of the polymer. Additionally, at a low concentration of 1–10%, the polymers were transparent with a bit of haziness in appearance. When the concentration was increased, drug crystals were observed on the top surface of the polymeric matrix, which resembles a phase separation. Microscope images revealed a thick layer of crystals embedded within the polymeric matrix with an extra amount on the top of it. This concentration was recorded to be higher than 17% for all the materials, and the Tg of the polymer was very hard to observe at that stage (Figure 6). The ability of the polymer to inhibit the crystal growth of a drug differs according to their affinity. This phenomenon was documented before by several authors [28,29,30,31]. However, no one has reported the impact of adding multiple drugs in a polymeric matrix.

**Figure 7:**
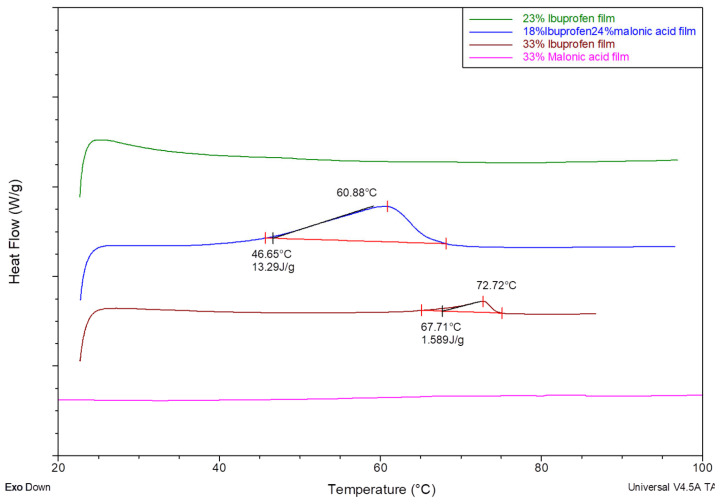
DSC thermogram of malonic acid, ibuprofen, and ibuprofen and malonic acid HPMCAS matrices.

Polymeric matrices containing dual drugs were opaque even with low concentrations of each drug. The DSC thermographs for mixtures of two drugs showed a clear endotherm for one of the drugs, which was not apparent in matrices with each individually. This was clear in that a lower concentration of ibuprofen showed a stronger endothermic peak, which reflects the crystallinity of the drug in a matrix containing only 18% ibuprofen in comparison to another containing ibuprofen alone at a concentration of 33% (Figure 7). Lower single-drug-loaded matrices revealed minimum to no existence of the endothermic peak of ibuprofen. It was also shown that the matrix degraded near the melting of malonic acid, which made it hard to detect using DSC analysis.

It is important to note that increasing the temperature above 110 °C caused the polymeric matrix to show a sign of degradation and confirmed that polymer degradation started at that level, which makes it hard to detect the melting endotherm for the malonic acid. To prevent machine damage, all scans were limited to 100 °C (Figure 7). Hence, scanning the other polymers for the melting point of flurbiprofen, naproxen, or malonic acid was not carried forward.

**Figure 8:**
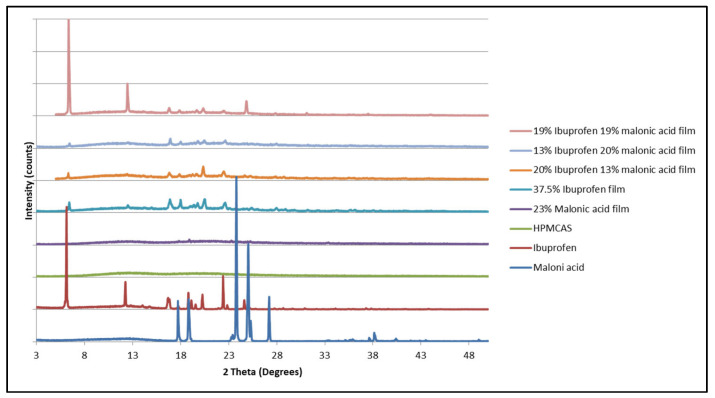
X-ray diffraction patterns of malonic acid, ibuprofen, malonic acid matrix, ibuprofen matrix, and blends of malonic acid and ibuprofen in polymeric matrices.

**Figure 9:**
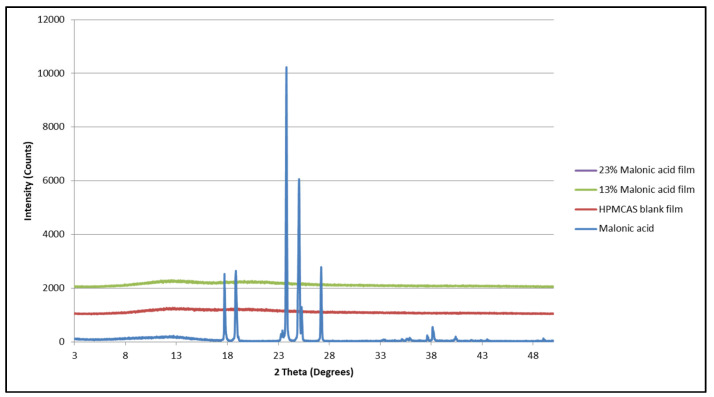
X-ray diffraction patterns of malonic acid, HPMCAS polymer, and polymeric matrices containing various concentrations of malonic acid.

Matrices containing mixtures of naproxen and malonic acid were not suitable for evaluation by DSC since the melting endotherm of the two compounds was higher than the onset temperature of polymer degradation. Hence, an X-ray diffractometer was used to assess the presence of the crystalline phase of the drug within the matrices. The diffraction peaks for all combinations in (Figure 8) that appeared in the polymeric matrices containing various concentrations of ibuprofen and malonic acid were at 2 theta of 6°, 12, 16, 18, 20, and 25. Similar concentrations of malonic acid in a polymeric matrix (without ibuprofen) did not show any diffraction pattern; rather, it showed an amorphous pattern (Figure 9). Although the 25° diffraction was close to that of malonic acid diffraction peak, it was weaker in intensity in comparison to that at 2 theta of 24° and 27°. Additionally, a similar peak has appeared in the diffraction pattern of the polymeric matrix containing 37.5% ibuprofen alone.

**Figure 10:**
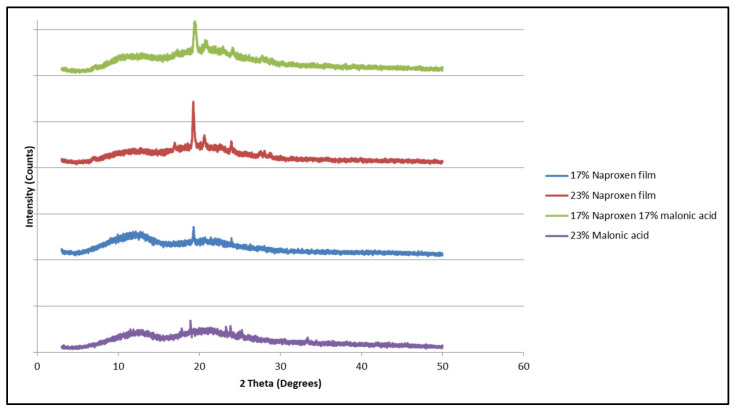
X-ray diffraction patterns of malonic acid, naproxen, and HPMCAS matrices containing each individually and matrices containing a mixture of both.

**Figure 11:**
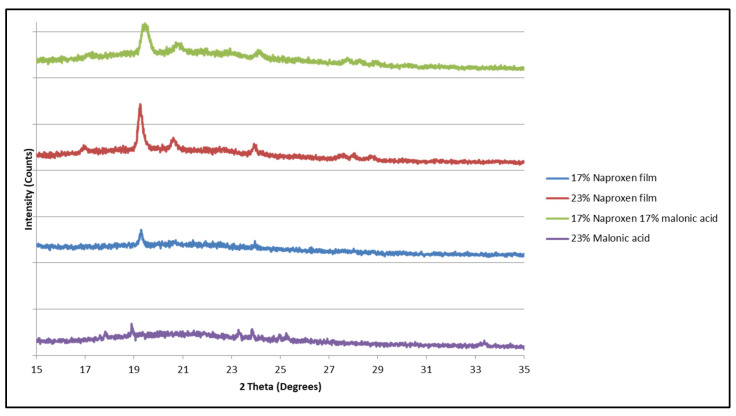
X-ray diffraction patterns of HPMCAS matrices containing naproxen, malonic acid, and both together. A magnification of (Figure 11).

A similar trend was observed in the diffraction pattern of polymeric matrices containing a mixture of naproxen and malonic acid. Only the naproxen diffraction pattern was visible in the matrix, and malonic acid seemed to remain as an amorphous material (Figure 10 and Figure 11) as the diffraction patterns visible were similar to that of matrices containing naproxen alone. 

In order to check that malonic acid is not the only compound that exhibits such behavior, flurbiprofen was selected based on its HSP value to that of the polymer (24.45 MPa^1/2^) (Table 2). A combination of naproxen and flurbiprofen in a polymeric matrix was tested, and the diffraction pattern showed diffraction peaks at 6°, 12°, 13°, 17°, 19°, and 23°, which are the peaks seen in the pure naproxen diffraction pattern.

It was noticed, though, that the diffraction pattern of the polymeric matrix containing flurbiprofen and malonic acid was similar to that of malonic acid alone, with low-intensity diffraction peaks at 17°, 19°, 23°, and 24° (Figure 14). These patterns are distinguished in the malonic acid diffraction pattern. Hence, it appears that flurbiprofen had higher affinity in this instance.

The presence of diffraction peaks equivalent to the pure drug was seen in mixed component dispersions that were not seen in a polymeric matrix containing a single drug at the same concentration, suggesting the combining of two compounds may be influencing the miscibility of one by the presence of the second one. This means that using the second drug (whether malonic acid or flurbiprofen) has worked as a blocker compound that reduced the saturation concentration of the polymeric matrix, and hence the second compound (whether naproxen or ibuprofen) has emerged in the form of a crystalline material.

Hence, it can be concluded that the diffraction peak of the polymeric matrices containing a mixture of drugs has resulted in one drug (malonic acid) being diffused within the polymer as an amorphous drug and the other has crystallized and phased out (separated from the matrix). The intensity of the peaks revealed that the amount of the crystalline phase was stronger than matrices containing higher concentrations of ibuprofen alone.

**Figure 12:**
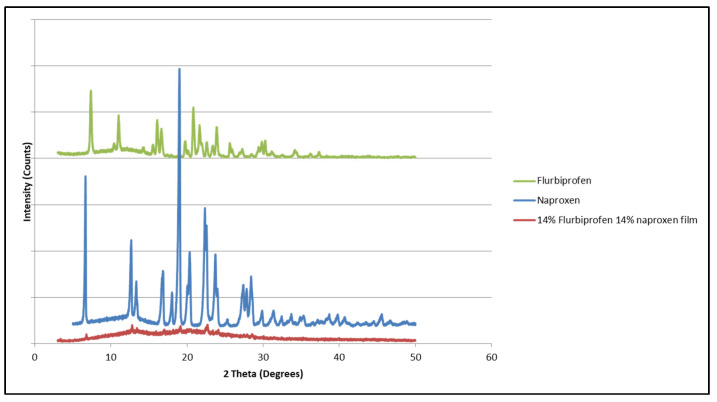
X-ray diffraction pattern of flurbiprofen, naproxen, and a polymeric mixture containing both.

**Figure 13:**
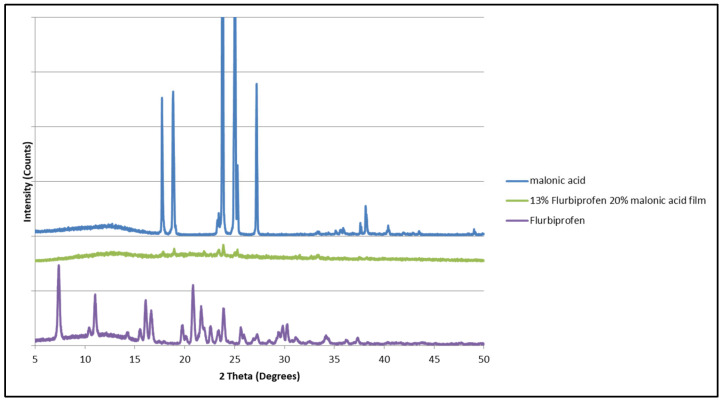
X-ray diffraction patterns of malonic acid, flurbiprofen, and a polymeric matrix containing both.

According to the solubility parameters presented in Table 2, it was noticed that malonic acid has a HSP value that is the closest to that of HPMCAS (22.47 and 24 MPa^1/2^, respectively). Here, malonic acid had a higher affinity to the polymeric matrix (interaction) than the other compounds. Also, when adding naproxen and flurbiprofen to the same matrix, it was observed that naproxen with the HSP value of 21.9 MPa^1/2^ had less affinity to interact with the polymeric matrix, which can be seen by the presence of its diffraction peaks (crystalline state) in comparison to that of flurbiprofen, which showed an amorphous presence. Flurbiprofen can be seen to have a closer value than that of naproxen. where from the analysis, it seemed that the material with a value closer to that of the polymeric matrix is the one that has the highest affinity to interact with it (Figure 12). To prove this, another combination containing malonic acid and flurbiprofen was prepared, where it was seen that the diffraction pattern of the matrix containing the two materials had a similar pattern to that of malonic acid, confirming that flurbiprofen had more affinity to interact with the matrix (Figure 13).

**Figure 14:**
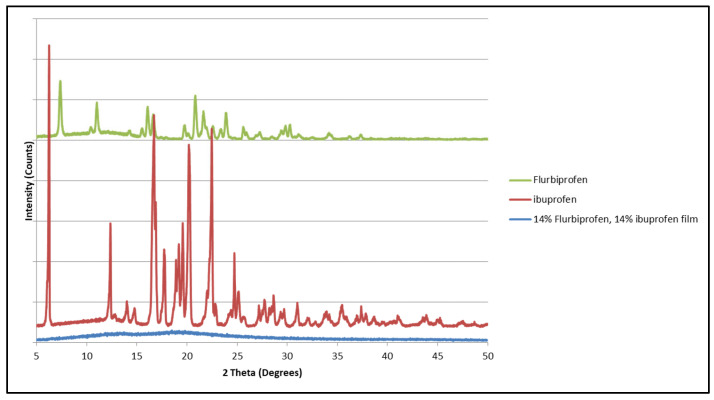
X-ray diffraction pattern of ibuprofen, flurbiprofen, and HPMCAS polymeric matrix containing both.

It was noticed that a polymeric matrix containing 14% flurbiprofen and 14% ibuprofen (Figure 14) did not show any distinguished diffraction pattern of either compound. This could be a result of a low concentration of these two compounds or a low blocking effect, which requires a higher concentration to have an effect. Further investigations are required with a higher concentration.

Two compounds (liquids or solids) were considered soluble when their solubility parameter values were no more than 7 MPa^1/2^ apart. There is growing literature covering the interaction between a single drug and a polymeric matrix. However, there are limited studies on the effect of dual drugs dispersed in a polymeric matrix. However, results obtained from an X-ray diffractometer and hot stage microscopy on dual drugs dispersed in the cellulosic matrix revealed the miscibility of one drug and the conversion to amorphous form but not the other, as a clear crystalline structure was noticed forming related to one of the drugs, despite using compounds with solubility parameter values that follow the rule that makes them miscible in the polymeric matrix. The evidence on drug solubility was further confirmed by preparing polymeric matrices with drugs separately proven to be soluble. Hence, it can be said that they were competing with each other when added together to the same matrix. Calculations showed that the drug with a smaller difference in solubility parameter value relative to that of the polymer was the miscible one, and the other with the greater difference in solubility parameter value relative to the polymer was the non-miscible one. It was noticed that malonic acid, which appears to have a HSP of 22.47 MPa^1/2^, which is closer to that of the polymer 24, revealed a higher affinity to interact and become miscible with HPMCAS than ibuprofen (19.5 MPa^1/2^) and naproxen (21.9 MPa^1/2^). The higher affinity was detected by the conversion of malonic acid into an amorphous form and the detection of the two later crystals. On the other hand, using flurbiprofen (24.45 MPa^1/2^) confirmed the theory by reducing the interaction affinity of malonic acid, which was detected by its crystals that formed at a lower concentration than matrices containing malonic acid alone. This confirmed the theory that the material with the closer HSP value to that of the polymer will have a higher affinity to interact with the polymer and reduce the potential for other compounds to interact with it.

## 5. Conclusions

It has been noted that HPMCAS was able to inhibit the crystal growth of the four drugs selected in this experiment: malonic acid, ibuprofen, naproxen, and flurbiprofen. The dispersion system formed was glass solution (amorphous in an amorphous system). Increasing the concentration of these APIs above a certain limit resulted in the appearance of API crystals as a result of phase separation, and large crystals were formed. When two APIs were added to the polymeric matrix, the API with the higher affinity seems to interact and form a solid solution with the polymeric matrix (amorphous dispersion) in comparison to the other, which appears to have a lower saturation concentration that manifested by the appearance of the crystals at lower concentration than for matrices with the API alone. The solubility parameters of Hildebrand seem to reflect this affinity, as the closest API HSP to that of the polymer is the one that interacts with the polymer and reduces the saturation concentration of the other API. This use of the HSP parameters could have the potential to predict the behavior of solid dispersions with multi-components.

The concept of solubility and cohesion parameters has been shown to discover the miscibility between two liquids as depicted by Hildebrand and later Hansen. In addition, this concept has been extrapolated to involve a solid dispersion system to determine the interaction between a single drug and a polymeric matrix. This research can also be extrapolated, if applied correctly, to predict the interaction between a polymeric matrix and a drug in a 3-component system.

## Figures and Tables

**Table 1:** Polymeric matrices containing the mixtures of a single and dual drugs in HPMCAS polymeric matrices.

Drugs and Drugs Mixtures in HPMCAS Polymeric Matrices	Mixing Ratios Drug w% in Total Matrix
Malonic acid	1%, 9%, 13%, 23%, 33%
Ibuprofen	1%, 9%, 13%, 23%, 37.5%
Malonic acid, Ibuprofen	19%, 19% 13%, 20% 20%, 13%
Naproxen	13%, 17%, 23%
Flurbiprofen	29%
Naproxen, Malonic acid	14%, 14% 17%, 17%
Flurbiprofen, Malonic acid	13%, 13% 13%, 20%
Flurbiprofen, Naproxen	13%, 20%
Flurbiprofen, ibuprofen	13%, 13%

**Table 2:** Hansen solubility parameters as calculated by Equation (2).

Compound	Solubility Parameter
Ibuprofen	19.5
Naproxen	21.9
Malonic acid	22.47
HPMCAS	24
Flurbiprofen	24.45

## Data Availability

Data are available upon request.

## Supplementary Materials

The following supporting information can be downloaded at: https://www.mdpi.com/article/10.3390/mps8010004/s1, Figure S1: TGA thermal degradation profiles of polymeric films containing 0%, 5%, 9%, 17% and 23% naproxen; Figure S2: TGA thermal degradation profiles of films containing 2% flurbiprofen and 29% flurbiprofen; Figure S3: TGA thermal degradation profiles for films containing 13% flurbiprofen—13% malonic acid and 13% flurbiprofen—17% malonic acid.
