# Supplementary material for: Evaluation of Drug–Polymer and Drug–Drug Interaction in Cellulosic Multi-Drug Delivery Matrices
Source: Methods Protoc. 2025 Jan 6;8(1):4. doi: 10.3390/mps8010004 (PMC11755489; doi:10.3390/mps8010004)
Supplement: Supplementary file 1 [file mps-08-00004-s001.zip › mps-3136448-supplementary.pdf]

Supplementary Data

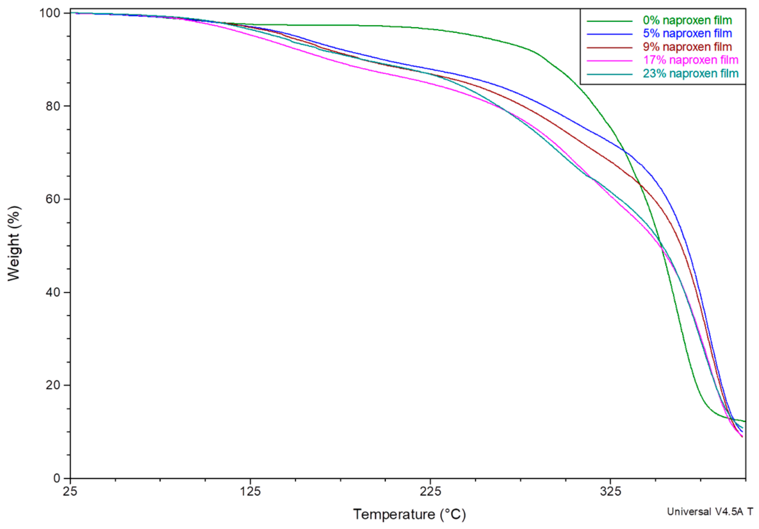

Figure S1. TGA thermal degradation profiles of polymeric films containing 0%, 5%, 9%, 17% and 23% naproxen.

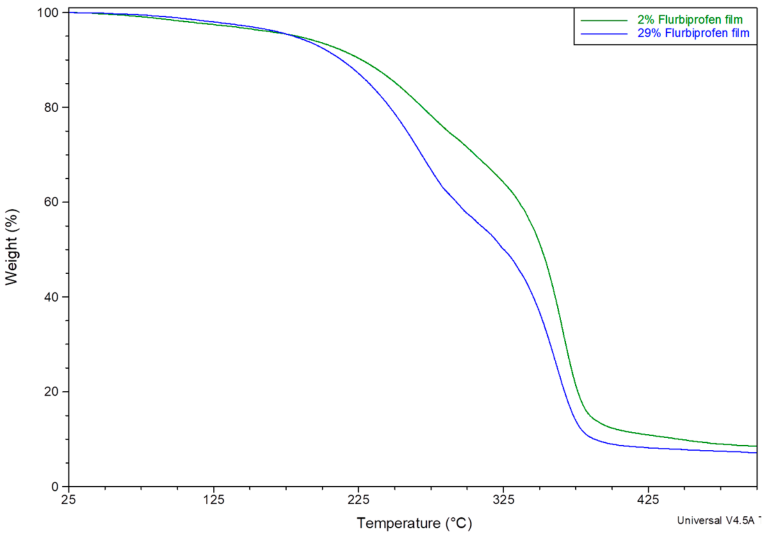

Figure S2. TGA thermal degradation profiles of films containing 2% flurbiprofen and 29% flurbiprofen.

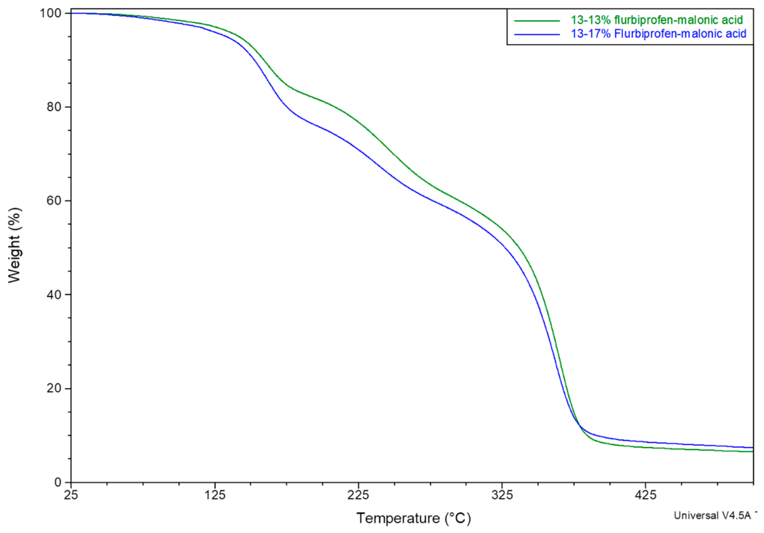

Figure S3. TGA thermal degradation profiles for films containing 13% flurbiprofen - 13% malonic acid and 13% flurbiprofen -17% malonic acid.
